# 4D‐PET data sorting into different number of phases: a NEMA IQ phantom study

**DOI:** 10.1120/jacmp.v10i4.2917

**Published:** 2009-10-28

**Authors:** Pietro Mancosu, Roberto Sghedoni, Valentino Bettinardi, Federica Fioroni, Mark A. Aquilina, Elisa Grassi, Ferruccio Fazio, Giovanni Borasi, Maria C. Gilardi

**Affiliations:** ^1^ Radiotherapy Department IRCCS Istituto Clinico Humanitas Rozzano (Mi) Italy; ^2^ Medical Physics Department Arcispedale S. Maria Nuova Reggio Emilia Italy; ^3^ Nuclear Medicine Department Scientifc Institute H San Raffaele Milan Italy; ^4^ Faculty of Medicine University of Milano‐Bicocca Milan Italy; ^5^ IBFM‐CNR Institute for Molecular Bioimaging and Physiology Milan Italy

**Keywords:** positron emission tomography, breathing inducing motion, 4D‐PET, motion compensation, image quality

## Abstract

This study aims to evaluate the dependence of 4D‐PET data sorting on the number of phases in which the respiratory cycle can be divided. The issue is to find the best compromise to reduce the conflicting effects induced by increasing the number of phases: lesion motion on each set of images decreases, but on the other hand image noise increases. The IQ NEMA 2001 IEC body phantom was used to simulate the movement of neoplastic lesions in the thorax and abdomen, investigating the effect of: target size (10 37 mm), lesion‐to‐background activity concentration ratio (4:1 and 8:1), total acquisition time (3, 6, 12, 20 min), and number of phase partitions (1, 2, 4, 6, 8, 10, 13). The phantom was moved in a cranial‐caudal direction with an excursion of 25 mm and a period of 4.0 sec. Five parameters associated to lesion volume and activity concentration were considered to assess the capability of the 4D‐PET technique to “freeze” the phantom motion. The results for all the parameters showed the capability of the 4D‐PET acquisition technique to “freeze” the lesion motion. The division into six phases was found to be the best compromise between temporal resolution and image noise for the phase where the “lesions” move faster; whereas the partition into four phases could be used if a stable breathing phase is considered.

PACS number: 87.57.uk; 87.57.cp

## I. INTRODUCTION

Positron emission tomography (PET) and more recently integrated PET/computed tomography (CT) systems are commonly used for tumor diagnosis, staging, restaging, and radiotherapy (RT) planning. Usually PET data are recorded over several minutes and, thus, the study must be performed in a free breathing condition. During the scan, various full breathing cycles are performed by the patient and, consequently, the reconstructed PET images are degraded by the internal motion induced by breathing, especially in the case of thorax and upper abdomen organs.^(^ ^1^ ^–^ ^2^ ^)^ These movements lead to a spread of the activity distribution – in particular, for the focal lesions – resulting in inaccurate radioactivity concentration quantification (i.e. standardized uptake value (SUV)),^(^ ^3^ ^–^ ^4^ ^)^ and in an erroneous estimation of the lesion shape and volume.^(^ ^5^ ^–^ ^6^ ^)^ Moreover, in the integrated PET/CT scanners, the different acquisition time of the two studies (CT scan is performed in a few seconds while PET data are collected for several minutes) can result in spatial mismatch, with artifacts being produced on PET reconstructed images when CT images are used for attenuation correction.^(^ ^3^ ^–^ ^4^ ^)^

More recently, respiratory gated four‐dimensional (4D) PET/CT acquisition techniques have been proposed to reduce the unwanted blurring on PET images and to improve the spatial matching between PET and CT images.^(^ ^6^ ^–^ ^7^ ^)^ Briefy, a 4D‐PET/CT acquisition protocol consists of a 4D‐CT and a 4D‐PET acquisition synchronized to the patient's breathing cycle. After the acquisition, both 4D‐CT and 4D‐PET data are sorted, divided and processed to generate new sets of images (phases), each of which is representative of a specific moment of the patient's respiratory cycle. As a result of this data processing, an improvement of the image quality and the quantitative accuracy of the tracer concentration estimation should be obtained by the compensation of the motion effects.^(3)^ On the other hand, the effect of such data processing results in highly noisy PET images, because each new set of data contains only a fraction of the total counts acquired during the whole 4D‐PET acquisition process.^(8)^ Therefore the options are to choose between a short frame duration (i.e. many phases) which would be noisy but would exhibit higher temporal resolution, or a longer frame duration which would be statistically superior but would exhibit a lower temporal resolution.^(^ ^7^ ^,^ ^9^ ^)^ Within these constraints, different acquisition schemes could thus be developed taking into account as well the specific characteristics of the organ under investigation.

In this work, we have analyzed the dependence of the image quality of a PET phantom as a function of the number of phases in which the 4D‐PET data can be sorted and divided, with the aim of finding the best compromise between the number of acquired events for each frame and the negative effects induced by the motion on the PET image quality.

## II. MATERIALS AND METHODS

### A. Acquisition protocol

In this study, the IQ NEMA 2001 IEC body (IQ‐N) phantom was used. It consists of a container with six spheres of different sizes (internal diameters: 37, 28, 22, 17, 13 and 10 mm).

The study was performed on the integrated system Discovery‐STE (D‐STE) (GE Medical System, Milwaukee, USA). The IQ‐N phantom tank and the spheres were filled with a homogeneous solution of water and F18. Two independent experiments were performed with different lesion‐to‐background ratios: (1) a ratio of 8:1 (32 kBq/cc to the six spheres and 4 kBq/cc to the background) to simulate lung lesions, and (2) a ratio of 4:1 (12 kBq/cc to the six spheres and 3 kBq/cc to the background) to simulate abdomen lesions. The activity concentrations were set to obtain count rates (prompt events) similar to those found in clinical studies (around 400–500 kcps).

In both experiments, the IQ‐N phantom was moved using the Respiratory Gating Platform system (Standard Imaging, Middleton, USA) to simulate typical motion caused by breathing. In particular, the maximum excursion was set to 25 mm in the cranial‐caudal (C‐C) direction in a 4.0 sec period. This time interval was selected because the mean breathing period during a 4D‐PET/CT acquisition protocol involving over 109 patients was found to be 4.0 sec (±1.0sec).^(10)^

The IQ‐N phantom motion was monitored by the respiratory gating system RPM (Real Time Position Management ‐ Varian Oncology System, Palo Alto, CA‐USA).

The acquisition protocol consisted of the following steps:

#### Phantom at rest

(1) CT Scout: (120 kV, 10 mA) to center the six spheres of the phantom.
(2) Rest‐CT: (Axial acquisition mode, 150 mA, 140 kVp, 0.5sec/rotation,beamcollimation=20mm,slicethickness=2.5mm,imagematrix=512×512pixels,fieldofview(FOV)=50cm). The Rest‐CT images were used for attenuation correction of rest‐PET data.
(3) Rest‐PET: (3D static acquisition mode, single axial FOV centered on the spheres, acquisitiontime=10min,attenuationcorrection=Rest‐CTfilteredtothePETresolution,3.27mm/slice,reconstructionalgorithm=3D‐OSEM,28subsets,2iterations,imagematrix=256×256 pixels, FOV=70cm).

#### Phantom moved using the Respiratory Gating Platform system

(4) CT Scout: (120 kV, 10 mA)
(5) 4D‐CT: (Cine modality, 150 mA, 140 kVp, 0.5sec/rotation,beamcollimation=20mm,slicethickness=2.5mm,imagematrix=512×512 pixels, FOV=50cm).^(^ ^3^ ^,^ ^11^ ^–^ ^13^ ^)^
(6) 4D‐PET: (3D acquisition mode, single axial FOV centered on the spheres, acquisitiontime=20min, list mode technique).^(^ ^8^ ^,^ ^14^ ^)^ During the 4D‐PET scan, the RPM system provided a trigger to the scanner at each maximum end‐inspiration position.

### B. 4D‐CT and 4D‐PET data sorting and reconstruction

The 4D‐CT images were sorted into 20 phases for subsequent volume calculation (4D‐CT series), and into 1, 2, 4, 6, 8, 10, and 13 phases for subsequent 4D‐PET attenuation correction (4D‐CTAC series).

**Figure 1:**
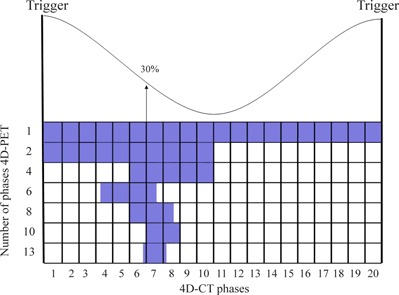
Schematic representation of the 4D‐PET list mode data sorting (1, 2, 4, 6, 8, 10 and 13 phases) used in this work. The 4D‐PET phase at 30% of the breathing cycle was used for the analysis of the data (highlighted).

The 4D‐PET list mode data between consecutive triggers were retrospectively sorted into sets of raw data, each of which represents a proportional percentage of the breathing cycle (phases). In particular, the PET list mode data were divided into 1, 2, 4, 6, 8, 10, and 13 groups of raw data (Fig. 1). The raw data of the same phase for each breathing cycle were then collected together. Furthermore, in order to evaluate the dependence from the total acquisition duration (i.e. statistics), the initial 3, 6, 12, and 20 min of the list mode data were considered for independent analysis.

The 4D‐PET raw data were random‐ and scatter‐corrected, compensated for attenuation by the corresponding 4D‐CTAC phases filtered to the PET resolution, and the data were then reconstructed using a 3D‐OSEM algorithm. The reconstruction parameters were: 28 subsets, 2 iterations, imagematrix=256×256 pixels, FOV=70cm.

### C. 4D‐PET data analysis

**Figure 2:**
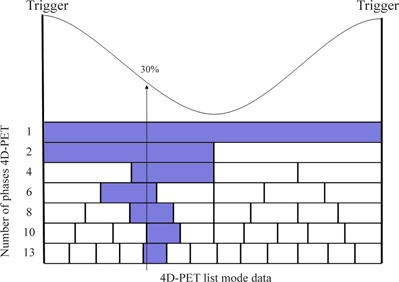
Schematic representation of 4D‐CT phases used for the calculation of volumes of each sphere in the 4D‐PET series. For each sphere, the lesion volume was calculated by combining the contours drawn on the 4D‐CT phases matching the 4D‐PET phase at 30% of the breathing cycle (highlighted).

A quantitative analysis was performed on the 4D‐PET series by considering the following five parameters:

(1) Lesion Extension $(L_{\text{Ext}})$, defined as the number of slices in which the lesion activity is visible over the background. In order to compare the different series of images, the same look‐up table was used to visualize the images. In particular, the minimum and maximum of the look‐up table were established on the rest‐PET series.
(2) Lesion Activity $(L_{\text{Act}})$, identified as the mean lesion radioactivity concentration. To calculate this parameter, an automatic approach was used in order to minimize involuntary subjective dependence. For each sphere of the IQ‐N phantom, the contour was drawn on all 20 4D‐CT phases. The lesion volume was then generated by using the Boolean operator “OR” on the sphere contours drawn on the 4D‐CT phases matching the 4D‐PET phases (as showed in Fig. 2) for the phase at 30% of the breathing cycle. A volume of interest (VOI) was generated on the 4D‐PET phases by applying the threshold of the maximum pixel value to achieve the 4D‐CT volume previously obtained. Finally, $L_{\text{Act}}$ was calculated as the mean concentration within the VOI.
(3) Lesion Volume *(LV)*, characterized by an automatic segmentation. For each sphere size on the PET rest series, the threshold of the parameter $L_{\text{Act}}$ was calculated, providing the true sphere volume. The same threshold was applied for each 4D‐PET series and *LV* was calculated as the voxels within the segmentation.
(4) Recovery Coefficient *(RC)*, expressed as
  (1) $$\frac{{\left(L_{\text{Act}}/B_{\text{mea}n}\right)}_{\text{mea}\text{sur}ed}}{{\left(L_{\text{Act}}/B_{\text{mea}n}\right)}_{\text{tru}e}}$$
  where $B_{\text{mean}}$ is the mean background concentration evaluated on 10 VOIs placed almost 50 mm away from the lesions and with a diameter equal to the correspondent IQ sphere.
(5) Lesion Over Background Variability *(LOBaV)* defined as

(2) $$\text{LOB}AV=\frac{{\left(L_{\text{Act}}-B_{\text{mea}n}\right)}_{\text{mea}\text{sur}ed}}{k\cdot \delta_{\text{Bac}\text{kgr}\text{oun}d}}$$

where *k* is a‐dimension value and $\delta_{\text{bac}\text{kgr}\text{oun}d}$ is the background standard deviation (assuming a Gaussian distribution). This parameter is based on the Rose model of statistical detection. By using the *LOBAV* parameter, the “net” signal $(L_{\text{Act}}-B_{\text{mean}})$ is compared with the background variability. In this work, *k* was set equal to 3.^(15)^

The full analysis was performed on the 4D‐PET phase at the 30% of the breathing cycle (Fig. 1), as the slope of the breathing curve is steepest at this point and thus the motion is fast. In addition, we considered in the analysis of the data the evaluation of the 50% phase of the 4D‐PET divided into four partitions only as necessary and interesting. This represents the most static point of the breathing curve.

The parameters were calculated also on the rest‐PET series to compare the 4D‐PET data with the static condition, assumed as reference.

## III. RESULTS & DISCUSSION

In this study, 352 different 4D‐PET series of images were reconstructed simulating different lesion‐to‐background activity concentration ratios (4:1 and 8:1), total acquisition times (3, 6, 12, 20 min), and number of phases (1, 2, 4, 6, 8, 10, 13) to assess the optimal 4D‐PET acquisition protocol in the case of neoplastic lung and abdomen lesions.

**Figure 3:**
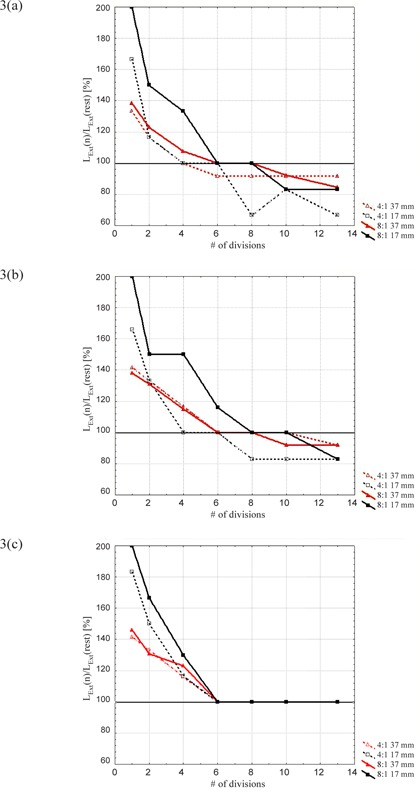
$L_{\text{Ext}}$ normalized to the rest‐PET series for the spheres of 17 mm and 37 mm diameter and for (a) 6‐min, (b) 12‐min, and (c) 20‐min reconstructions.

**Figure 4:**
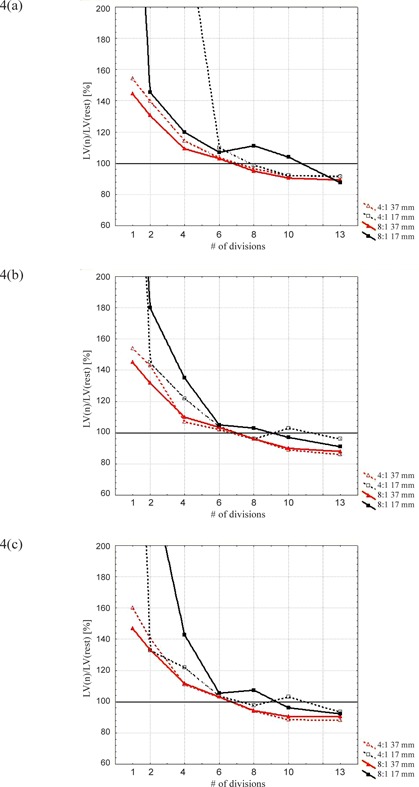
$L_{\text{Act}}$ normalized to the rest series for the spheres of 17 mm and 37 mm diameter and for (a) 6‐min, (b) 12‐min, and (c) 20‐min reconstructions.

**Figure 5:**
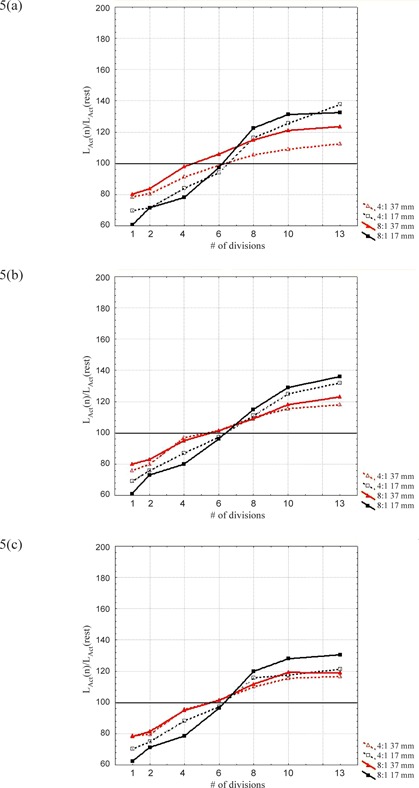
*LV* normalized to the rest‐PET series for the spheres of 17 mm and 37 mm diameter and for (a) 6‐min, (b) 12‐min, and (c) 20‐min reconstructions.

In Figs. 3–5, we report $L_{\text{Ext}},L_{\text{Act}}$, and *LV* calculated on the 30% 4D‐PET phases, normalized to the results obtained on the rest‐PET series for the spheres of 17 and 37 mm diameter and for both activity concentration ratios (4:1 and 8:1). The parameters were not evaluated for the 3‐min reconstructions, as the background was predominant and therefore accurate analysis could not be performed. The 3‐min 4D‐PET images were specifically analyzed by the parameter *LOBAV.*

The first parameter $(L_{\text{Ext}})$ was considered to quantify the capability to “freeze” the phantom motion since it evaluates the visible lesion length in the C‐C direction (i.e. the greater motion direction). In particular, an excursion length of 25 mm was considered along the C‐C direction. This value overestimates the usual lesion motion induced by breathing, as the 4D‐PET gating techniques were developed for highly‐mobile lesions. (For motionless lesion, the 4D tools is not necessary.)

In all cases $L_{\text{Ext}}$ decreases by increasing the number of phases and becomes equal to the result of the rest‐PET series for the sorting into six phases (Fig. 3). The overestimation of lesion size/volume for small number of phase partitions (1, 2 and 4) demonstrates incomplete motion compensation. For the 20‐min reconstructions (with high statistic data), $L_{\text{Ext}}$ remains stable;however, in the case of 12‐ and 6‐min reconstructions, $L_{\text{Ext}}$ is underestimated as the total number of phases is increased. This effect could be related to a noise effect that depends (assuming a Poisson distribution) on the inverse square root of the total number of prompt events (i.e. statistics) and, therefore, it becomes significant for the higher partitions and lower acquisition times. (For example, the 3‐min 4D‐PET acquisition time divided into 13 phases means a statistic of only 12 sec.) The parameter $L_{\text{Ext}}$ was defined as the number of slices demonstrating a signal higher than background. When the background increases, the cutoff (i.e. threshold) has to rise, and thus some lesions or parts of the lesions could be lost.

The next parameter, $L_{\text{Act}}$, evaluates the mean activity concentration within the volume of lesion motion. This volume is specifically calculated for each 4D‐PET partition using the 4D‐CT images, as showed in Fig. 2.

$L_{\text{Act}}$ increases with a rising number of breathing divisions (Fig. 4). The partition which matches best the value of the rest series is the division into six phases. Underestimation of the radioactivity concentration for less than six partitions is attributed to incomplete motion compensation. Overestimation for more than six phases could be ascribed to the behavior of the iterative reconstruction algorithm which, in the case of low counting statistics, could lead to lesions activity overestimation, as shown by Reilhac et al.^(16)^

The parameter *LV* was included to assess the capability to “freeze” the phantom motion and to achieve the true sphere volume, calculated as

(3) $$V_{\text{tru}e}=\frac{4\pi }{3}\cdot r^{3}$$

where *r* is the sphere ratio reported by the IQ‐N phantom data sheet, from the 4D‐PET phases.

*LV* shows a similar shape obtained for $L_{\text{Ext}}$ as reported in Fig. 5, confirming the previous analysis. This result reveals the adequacy of the automatic technique based on thresholding and, indirectly, also of the $L_{\text{Act}}$ evaluation. In particular, a specific threshold was necessary for each sphere dimension to take into consideration the partial volume effect (especially for small lesions). The choice was to calculate these thresholds on the rest series since the spheres volumes are known from beforehand in this situation; thus a threshold of the mean activity can be evaluated. The mean value of the radioactivity concentration was preferred to the maximum pixel value as less sensitive to statistical fluctuations that become dominant for the higher partitions.

The fourth parameter, *RC*, is representative of radioactivity concentration accounting for background effect and normalized to the corresponding values of the rest series in order to minimize the partial volume effect.

**Figure 6:**
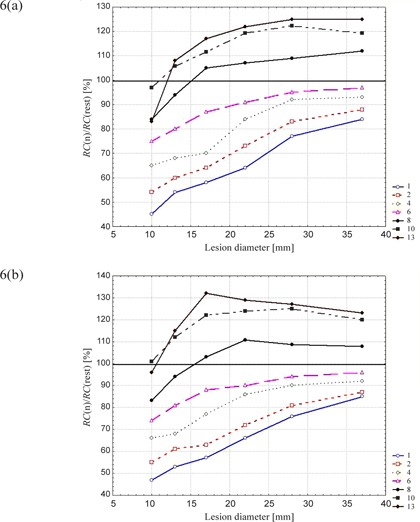
*RC* in function of lesion diameter normalized to the rest‐PET series for 20‐min reconstructions and for activity concentrations ratios of (a) 4:1 and (b) 8:1.

In Fig. 6 we demonstrate *RC* values normalized to the results of the rest series as a function of lesion size for all the partitions and of both the radioactivity concentration ratios (4:1 and 8:1) for the 20‐min scan. Normalized *RC* values increase with lesion size, and the curve closest to a normalized *RC* value of 1 is that corresponding to division into six phases. However, even in this case, when considering the dependence on lesion size (Fig. 6), an incomplete recovery of the activity concentration is observed for small spheres. A reason for this could be related to the relative motion of spheres. In fact, the relative motion was 250% of the sphere size for the 10‐mm lesion (the excursion motion was set to 25 mm in the C‐C direction), while the relative motion was 68% for the 37‐mm sphere. Another reason for this is the fact that the voxel thickness is 3.75 mm.

The last parameter, *LOBAV*, measures lesion detectability, accounting for background standard deviation (i.e. image noise).

In the case of the 17 mm sphere size, the highest value for the parameter *LOBAV* was obtained for ratio of 8:1, 2 phase partition and 20 min (Table 1. In all cases, the 8:1 values have higher detectability than the 4:1 cases, indicating that lesion‐to‐background ratio plays an important role in revealing the lesion. Moreover, *LOBAV* increases with total acquisition time (i.e. statistics) and decreases with the number of partitions. This result shows the leading role of noise. In any case, the value was greater than 1 even in the ‘worst’ condition, revealing the possibility to detect the 17‐mm lesion. Table 1 can be analyzed by looking at the diagonal values, as well. In this way, the data come by sets of images with the same statistics, and thus noise dependence is eliminated. For example, the 4D‐PET data divided into 1 phase and with total duration of 3 min has the same statistics of 2 phases and 6 min (6min/2phases=3min), of 4 phases and 12 min, and almost the same statistics of 6 phases and 20 min. The parameter increases for all the diagonals, revealing the capability of the 4D‐PET phase partition to take account for motion.

The last part of this investigation was focused on the variations that arise when considering all different phases of the same partition. The analysis was performed on the phase at 50% of the breathing cycle (i.e. end‐expiration, which is the most stable phase) to compare the two “extreme” moments of the breathing cycle, when the curve slope is maximum and when the mobility is lowest. In particular, the partition into four phases was analyzed as the six phases were found to be the best compromise to freeze the lesion motion on the phase showing the higher excursion.

Table 2 reports the $L_{\text{Ext}}$ values for the partition into four phases (at 30% and 50% of the breathing cycle) normalized to the rest series for all the sphere sizes. Only the 20‐min reconstruction was analyzed and resulted as less influenced by the noise.

As can be seen from Table 2, the phase at 50% shows complete motion compensation; on the other hand, this does not happen for the phase at 30%. This is a result of the relatively lesser amount of sphere motion on the intraphase time (i.e. the phase at 30% includes all the prompt events in the range 25%–50% of the breathing cycle; the phase at 50% derives from the range 50%–75%). This analysis suggests that the most stable phase to quantify the lesion activity should be used. As for the phase at 50%, the division into six phases does not give more motion information; on the contrary, the noise increases with respect to four phase partitions.

The authors point out that the analyses have been performed using a specific PET/CT system, and different results could arise using different scanners and different acquisition parameters. Furthermore, equispaced bins have been used to sort the list mode data. Non‐equispaced bins could result in different outcome. For example, collecting together the most stable breathing parts could reduce noise effect without increasing motion distortion, and thus could increase the lesion detectability.

Finally, we would like to stress the fact that the results of this evaluation are pertinent only for 4D‐PET and cannot be applied for 4D‐CT acquisition technique due to the different 4D data sorting. For 4D‐CT data sorting, Pan et al.^(12)^ and Rietzel et al.^(13)^ recommend a large number of reconstructed images per bed position (more than 10). To this purpose, in the present analysis, the volumes were determined dividing the 4D‐CT images into 20 phases.

**Table 1:** LOBaV calculated for the sphere of dimension 17 mm; lesion‐to‐background ratios of (a) 4:1; (b) 8:1.

*(a) 4:1*	*3 min*	*6 min*	*12 min*	*20 min*
1 phase	2.0	3.1	3.7	4.2
2 phases	1.7	2.5	3.3	3.9
4 phases	1.6	2.2	3	3.7
6 phases	1.6	2.5	3.3	3.8
8 phases	1.6	2.2	3.2	3.7
10 phases	1.4	2.1	3.3	3.8
13 phases	1.3	2.0	3.1	3.7
*(b) 8:1*	*3 min*	*6 min*	*12 min*	*20 min*
1 phase	4.1	5.5	6.5	7.7
2 phases	4.0	5.2	6.0	8.4
4 phases	3.7	5.0	6.4	7.8
6 phases	3.8	4.9	6.2	7.9
8 phases	3.6	4.9	6.0	7.8
10 phases	3.5	4.2	5.0	7.5
13 phases	2.9	4.2	4.5	7.3

**Table 2:** $L_{\text{Ext}}(4\;\text{phases})/L_{\text{Ext}}(\text{rest})$ for all the spheres sizes and lesion‐to‐background ratios of 4:1 and 8:1.

*Sphere size (mm)*	*37*	*28*	*22*	*17*	*13*	*10*
4:1 50%	1.0	1.0	1.0	0.8	1.0	1.0
4:1 30%	1.2	1.1	1.4	1.2	1.4	1.3
8:1 50%	1.0	1.0	1.0	1.0	1.0	1.0
8:1 30%	1.3	1.2	1.3	1.3	1.2	1.3

## IV. CONCLUSIONS

By making use of the list‐mode flexibility (i.e. the ability to divide the acquisition data according to preference, both in terms of number of phases and acquisition duration),^(^ ^8^ ^,^ ^14^ ^)^ an IQ‐N phantom was acquired in motion, simulating a breathing cycle, in order to study the image quality as function of the number of phases in which a 4D‐PET study is divided. Our results showed that partition into six phases seems to represent the best overall compromise between motion compensation and the effect of image noise. The partition into four phases could be used if a stable breathing phase is considered.
